# Travel Behaviour of Vulnerable Social Groups: Pre, during, and Post COVID-19 Pandemic

**DOI:** 10.3390/ijerph191610065

**Published:** 2022-08-15

**Authors:** Nima Dadashzadeh, Taimaz Larimian, Ulysse Levifve, Rok Marsetič

**Affiliations:** 1 Intelligent Transport Cluster, Faculty of Technology, University of Portsmouth, Portsmouth PO1 3HF, UK; 2 School of Architecture, Building and Civil Engineering, Loughborough University, Loughborough LE11 3TU, UK; 3 Civil Engineering Faculty, Technical University of Compiègne, 60200 Compiègne, France; 4 Faculty of Civil and Geodetic Engineering, University of Ljubljana, 1000 Ljubljana, Slovenia

**Keywords:** COVID-19, pandemic, travel behaviour, mobility, transport, vulnerable social groups

## Abstract

Since the emergence of COVID-19, travel restrictions due to the pandemic have influenced several activities, in particular the mobility patterns of individuals. Our main goal is to draw the attention of scholars and policy makers to a specific segment of the population, namely (1) older people, (2) persons with disabilities (PwDs), (3) females, and (4) low-income population that are more vulnerable for travel behaviour change due to crisis such as the COVID-19 pandemic. This article systematically reviews the studies that have explored the implications of COVID-19 for the mobility and activities of individuals pre-, during, and post-pandemic using the PRISMA method. It is found that there are a few studies regarding the travel and mobility needs and challenges of older people and PwDs, and there is no direct study concerning female and low-income individuals while such crisis exist. Questions such as “What are the adverse impacts of restrictions on their travel behaviour?”, “How can they travel safely to work, shopping, and medical centres?”, “Which transportation modes can be more effective for them?”, and “What are the government and policy makers’ role in providing accessible and affordable mobility services in the presence of such crisis?” are without relevant answers in the literature.

## 1. Introduction and Background

On 11 March 2020, the World Health Organization (WHO) officially announced COVID-19 as a pandemic situation and called for essential protective measures to strengthen preventive hygiene such as wearing a mask in public places, elimination of physical contact, elimination of gatherings and large events, elimination of unnecessary travel, and implementation of quarantine/lockdown [1]. In addition to the first version of COVID-19, new variants have appeared around the world such as Delta and Omicron [2]. Since 2020, various restrictive measures and policies were applied among different countries, all having significant implications for people’s mobility. For instance, overall mobility in Spain fell by more than 75% when the COVID-19 outbreak was introduced [3]. In Poland, a significant decrease in total travel time during the outbreak was observed regardless of travelers’ age and gender [4]. Following the state restrictions, multiple private companies provided their employees the option of home-based work, reshaping daily commuting patterns, although different clusters of workers can have different attitudes towards working from home [5]. Concerns about possible virus infection also influenced mode choices, particularly in favour of private modes [6]. Specifically, active mobility increased significantly during the outbreak combining compliance to the restriction measures and lower risk of infection with the opportunity for outdoor exercise [7,8,9,10,11,12]. Public transport use dropped by almost 80–90% in major cities in China, Iran, U.S., Italy, Spain, and France [13,14], mainly because of people’s perception of the increased risk of infection in public transport, but also due to operation cuts during the pandemic [15,16,17]. Shared mobility systems were also perceived as a high-exposure mode to the virus resulting in lower levels of usage, although bike- and scooter-sharing companies were influenced to a lesser extent than ride-hailing or carpooling services [18]. The COVID-19 pandemic has created new or further attenuated mobility and activity restrictions that vulnerable social groups (VSGs) such as older people, people with mental and physical disabilities, low-income population, and females were already experiencing before the outbreak. For example, older people limited their activity in much higher rates than other age groups in terms of time and distance spent outside, while their mobility capital was severely restricted due to limited access to a car (or being chauffeured) and the avoidance of PT as a high-exposure mode to the virus [7,15,19,20]. People with visual and mobility impairments faced significant difficulties in visualizing and keeping social distances, respectively, being exposed to higher health risks, while paratransit services operation cuts contributed to further isolation of these social groups [21,22]. People on low-income appeared less flexible in reducing their amount of travel through home-based working, while they continued using public transport during the pandemic at higher rates than people with higher incomes [10,15,23]. Moreover, females tended to take over increased housework and caring activities (e.g., of children and older people) during the pandemic, negatively affecting their ability to keep-up with their job activities [6,24,25,26]. The implications of COVID-19 for the mobility and activities of VSGs have attracted less attention in the scholarly literature compared to the respective changes of other social groups [6,7,11,16]. However, VSGs tend to be exposed to higher health risks, due to pre-existing medical and social conditions that influence their mobility and activity patterns.

To this end, this paper systematically reviews the studies that have explored the implications of COVID-19 for the mobility and activities of VSGs pre-, during, and post-pandemic when travel restrictions are lifted. The review focuses on the outcomes rather than on the methods of the studies and highlights broader resilience issues of urban and transportations systems with respect to supporting VSGs in times of crisis such as the COVID-19 pandemic. The rest of the paper is structured as follows. Section 2 describes the methods and data of the studies included in our literature review. Section 3 presents first the results regarding the geographic distribution of the studies and then the outcomes of our analysis of the studies focusing on the implications of COVID-19 for the mobility and activities of older people (Section 3.1), people with disabilities (Section 3.2), women (Section 3.3), and people on low income (Section 3.4). In Section 4, we present our conclusions per VSGs and discuss the associated policy implications.

## 2. Materials and Methods

**Figure 1:**
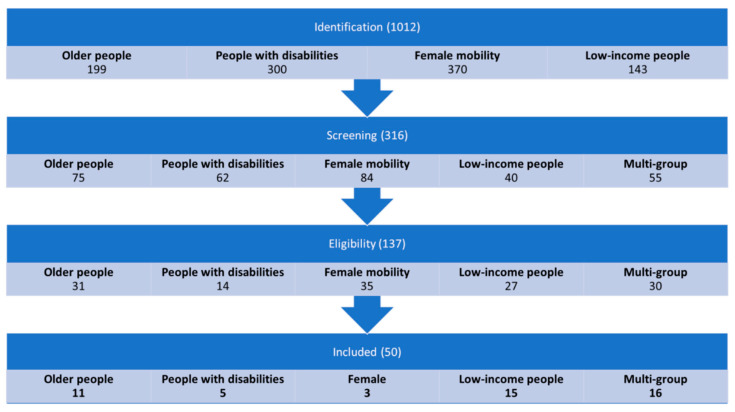
The four stages of the PRISMA protocol and the number of papers identified per stage.

We applied the PRISMA protocol (preferred reporting items for systematic reviews and meta-analyses) [27] to select the studies for our literature review. The PRISMA protocol is performed in four stages involving identification, screening, eligibility assessment, and, finally, inclusion of studies in the analysis (see Figure 1).

### 2.1. Search Strategy

In the first stage (identification), we used the following keywords and Booleans (“AND”) to identify the relevant studies. We searched for Web of Science, Scopus, and Google Scholar listed peer-reviewed articles, conference papers, and book chapters published from January 2020 to the writing time of the article (December 2021). The types of articles included original research, review articles, short reports, and case studies.

- COVID-19 AND older people;
- COVID-19 AND people with disabilities;
- COVID-19 AND gender, female, women;
- COVID-19 AND low-income people.

Our initial search returned a total of 1012 papers distributed to 199 papers on older people, 300 papers on people with disabilities, 370 papers on gendered-based mobility, and 143 papers on people on low income (see Figure 1).

### 2.2. Selection Strategy

During the screening stage (stage 2), selection was made with the title of the articles. We removed all the articles that have an out-of-scope title. The eligibility-check stage (stage 3) required an extensive reading of each abstract and checking whether or not the article directly or indirectly discusses the effects of COVID-19 on the mobility of vulnerable user groups. After the screening and eligibility check, we selected a total of 50 papers for in-depth review and inclusion in our analysis (11 papers on older people, 5 papers on people with disabilities, 3 papers on gendered-mobility, 15 papers on people on low income, and 16 studies that refer to more than one vulnerable social group). More detailed information regarding the studies included for the full review is presented in Table A1 (Appendix A).

## 3. Results

This section discusses COVID-19 studies’ findings concerning our target user groups, namely older people, persons with disabilities, females, and low-income people.

### 3.1. Older Adluts

Older people’s activity shifted during the outbreaks. Studies reported a decrease of older people’s overall mobility: less time spent outside, less distance travelled, and trips made in a smaller perimeter around their home [7,15,19] (Table 1). Although this change was common for all age groups when mobility restrictions were introduced, studies note particular reactivity of the older people compared to other age groups, such as a drop in activity of older people that happened earlier and a stronger drop in activity than the others [28]. Due to their higher vulnerability to the virus, older people were more co being infected by the virus and had more tendency to avoid crowed places, including PT. Moreover, trying to avoid attendance in places with other people, the modal share of older people showed a decrease in shared mobility as well [20]. Car and special transportation use by older people (such as community transport or paratransit) also decreased [15,29], with the latter being affected by mobility restrictions and having decreased service (reduction in number of vehicles) or deactivated service. Moreover, some older adults, being less likely to have a driver’s license, did not have someone else to drive them, thus reducing their overall mobility during the pandemic. The COVID-19 pandemic influenced the travel purpose of older people as well. They stopped travelling for leisure [30], and the main motives of their trip were going to groceries stores (common for all ages), pharmacies and newspaper stands [31].

### 3.2. People with Disabilities

The literature already explicated the difficulties of PwDs to access transportation systems whether it was before or during COVID-19 and revealed that the degree of disability of the person is directly linked to their time spent at home [41]. The pandemic outbreak made travel much more difficult for PwDs (Table 2). According to Beukenhorst et al. [41], daily time spent at home for people living with amyotrophic lateral sclerosis (ALS) (Amyotrophic lateral sclerosis (ALS) is a rare neurological disease that primarily affects the nerve cells (neurons) responsible for controlling voluntary muscle movement (source: https://www.ninds.nih.gov/amyotrophic-lateral-sclerosis-als-fact-sheet, accessed on 10 October 2021)) increased to almost 24 h, and the daily distance travelled dropped. Out in the street and in public spaces, it has also been reported that PwDs are experiencing difficulties to follow the required distances with other persons (e.g., people with visual impairment having troubles to visualize the distances and people with mobility impairment having trouble keeping distances) inducing higher exposure to the virus for PwDs when travelling [22]. The pandemic aggravated difficulties of PwDs to access PT and other shared mobility modes. Paratransit services known also as community transportation (in UK), a demand-responsive transportation (DRT) system that many PwDs depend on, dropped during the pandemic, causing incapability to travel for many disabled people [25]. In PT, PwDs reported getting less accessing assistance [21]. The difficulties to access transportation during the pandemic had a direct impact on their well-being, involving less access to medication, health care, and essential services [22]. PwDs were more likely to travel for medical reasons and to provide help to other vulnerable persons than other groups during the pandemic [41,42].

### 3.3. Gender Gap

There is a mixed picture regarding the impacts of the pandemic on the mobility and activities of different genders. Some studies reported a higher rate of overall mobility and longer trips for men compared to women [6,12,44], while other studies reported a higher drop in men’s overall mobility [26] (Table 3). Several studies identified an increase of car use and walking by women [10,11] and a decrease in PT, while there is also evidence of no significant change of mode choice during the pandemic between men and women [6]. Regarding trip motives, women reduced leisure travel (being more compliant with mobility restriction measures), grocery shopping, and work activities at a higher rate than men [23,44]. Moreover, studies reveal a higher vulnerability and an increased exposure of women to the virus related to (a) the higher tendency of women to use PT than men, exposing them to the infection risk [4]; (b) the type of jobs they hold (e.g., in Belgium, home care assistants are held by women up to 97%); (c) the unequal home-duties repartition that induce higher mobility for women than men in addition to their higher reliance on PT [24]; and (d) the higher needs of medical care for women such as prenatal care.

### 3.4. Low-Income People

Before the pandemic, mobility of low-income population (LIP) was described in the US by a lower number of trips per day, a higher share of PT, lower rate of car ownership, higher share of walking for shopping, and a higher commuting carpool rate compared to higher income groups [25]; however, carless LIP used ride hailing for essential trips more than carless high-income population (HIP) [46]. Evidence suggests that during the pandemic, the number of trips and the distance travelled by LIP has decreased less than those of the higher income groups, indicating that LIP were less flexible to reduce their mobility when mobility restrictions were introduced [25,47], mostly due to their unsuitable jobs for home-based work [17,44,48]. Evidence also suggests that even though LIP were concerned about the risk of infection, they tend to use PT more during lockdown [23,49] and motorized two-wheelers in the case of India [11]. The demand for using ride hailing (Uber, Lyft, etc.) before and during the pandemic has not changed for LIP [50]. The interest in buying a car after the pandemic was higher for LIP compared to higher income groups in China but did not differ across income groups in Europe and the U.S. [21]. Moreover, according to Chen et al. [25], LIP have slightly higher health care needs compared to the other income groups, and they are facing more transportation barriers to meet those needs, as they have a lower rate of car ownership and as PT service was affected by the mobility restriction measure. Therefore, COVID-19 emphasized the existing transportation accessibility inequity in addition to having amplified it between income groups. In response, some studies carried out on this topic show the importance of the active transportation in low-income countries and their positives outcomes, counting health, social, and climates benefits [51]. Table 4 presents the key findings of the reviewed studies on the pandemic and mobility of low-income people.

## 4. Conclusions and Policy Recommandations

It is a fact that equity issues such as the needs and challenges of vulnerable social groups have not been considered by most of the countries in the analysis, planning, and implementation of transportation and mobility services [58]. This can affect travel behaviour of VSGs compared to other user groups. Thus, countries should make mobility more resilient and accessible for everyone. In addition, the mobility of VSGs has also supposed to be influenced by mobility restriction measures applied during crisis such as the COVID-19 pandemic. Therefore, this study presents a systematic literature review on the pandemic impacts on mobility of VSGs. We had an extensive search among existing studies regarding the pandemic impacts on people’s mobility and tried to extract the most relevant findings considering VSGs. Considering the aim of our study, existing findings, research gaps, and directions are as follows:

**Older people**: With an increase of the aging population, older people are facing mobility issues and shifts from private cars to other shared modes. Being the most at-risk group of users for COVID-19, older people change their mobility habits (compared to before the pandemic) to avoid crowed places, PT, and shared-mobility modes. Many articles explore the change in mobility activity (i.e., time outside, distance travelled), but only one assesses some changes of older people’s mode preferences. It is known that older adults’ mobility patterns differ from those of younger age groups. The literature is well documented on the impacts of a pandemic on their activity, but very few explored the mode preference changes. Thus, one should evaluate their preferences and challenges for each transportation mode before, during, and after the pandemic.

**Persons with disabilities**: PwDs always faced strong difficulties accessing transportation systems because of the low inclusiveness of urban transports. Paratransit service and other ride-sharing modes are the main component of PwDs’ mobility as they rely on the assistance of other people. Only little information has been discussed by existing studies on how PwDs meet their needs during COVID-19 to commute. Moreover, the study areas are limited to the U.K. and U.S. To have a better understanding of PwDs’ mobility patterns in such crisis, it would be interesting to have evidence on their methods to meet their mobility needs and some evidence on their travel and mobility patterns in all continents. During the COVID-19 pandemic, PwDs experienced many difficulties to travel because of the diminution of PT and paratransit service and to ensure social distancing with others. Their travel activities have decreased drastically during the pandemic. On the other hand, transportation accessibility as a barrier has a direct impact on PwDs’ job accessibility [59]. COVID-19 mobility restriction revealed that the fermented availability of home-based work (tele-working) could be an opportunity for PwD to access new jobs. Therefore, one should assess how PwDs respond to their needs during such crises and how they reached their destination (workplaces, medical centres, shopping, etc.).

**Gender gap:** As a well-stablished fact, the travel and mobility pattern of women and men are different [60]. Men have a higher share of private transportation and women have more reliance on PT. During the outbreak, there was less decrease in women’s mobility activity in some countries due to their essential jobs and remarkable decrease in other countries due to a lower driver’s license ownership rate and PT deactivation. Another challenge that these group of users deal with during the pandemic is their medical needs such as prenatal care, incompatible with telemedicine. There is a lack of data on the change of travel behaviour for each mode. Furthermore, men’s behaviour does not vary much depending on the country, whereas women’s behaviour, even during the pandemic, is very different by country (e.g., Belgium and India). Therefore, one should study the impacts of such crisis on gender gap in mobility before, during, and after the pandemic and explore the response of different genders in different counties.

**Low-income population**: LIP have a higher share of PT use, walking for shopping, carpooling to commute because of the affordability of these modes, and a lower rate of car ownership. During the COVID-19 pandemic and due to the (deactivation) limited PT services, LIP experienced difficulties for transport as many of the jobs held by LIP are not suitable with home-based work. This paper recognized that travel patterns of low-income households are different than the other income-groups, and that the deprivation of PT during COVID-19 particularly affected their mobility habits. There is also a lack of information on the outcomes of pop-up active transportation facilities (e.g., bicycle, walking, etc.) on LIP’s travel behaviour. Those transportation modes have proven to be valuable to meet many LIP’s mobility needs. Consequently, one should analyze their needs and challenges due to mobility restrictions and explore how they meet their mobility needs during such crisis.

**Policy implications:**

- **Active Transport:** There is a link between safe bike lane or bike lanes with good connectivity to amenities with the increase of cycling activities [61]. In order to satisfy the need in active transportation during and post-pandemic (when mobility restrictions are lifted), implementing new cycling lanes and widening the pedestrian roads will improve the mobility of VSGs who are interested in active transport, in particular older people and persons with disabilities that encountered problems in keeping (1.5–2 m) social distances. Local authorities and policymakers should prepare contingency plans and guidelines for future emergencies where other modes of transport lose their capacity and efficiency. In such situations, people from different age-groups and with different physical abilities should be able to have access to alternative active transport options. Neighborhoods and urban built environments should become more people-friendly with more compact, diverse, and mixed land uses to encourage people to switch to active modes of transport.
- **Public Transport:** It is undoubtedly true that PT was the most negatively affected as there is a higher concern of the infection risk. There are two different PT systems around the world: owned and operated by private companies and owned and operated by local authorities/municipalities. During such crisis, bus and rail operators due to the collapse in revenue from ticket sales faced significant financial difficulties. Government or local authorities should provide these companies with public funds to ensure accessible and affordable PT services for VSGs. Apparently, more research is required about efficient business models, particularly if based on PPPs and subsidies for mobility services. This study emphasizes the importance of shifting governments and policymakers’ mindset towards a pandemic-focused governance. This includes adopting a more resilient mindset that is prepared for future emergencies that require governments and transport providers to shift public transport users to other modes of transport (e.g., ride sharing and bike sharing services). Another policy implication would be the ability of public transport providers to shift from fixed-hour services to more flexible services to be able to accommodate the needs of users in case of emergency.
- **Shared mobility:** Results of this study indicated that one of the main reasons for customers’ intention to avoid shared mobility during the pandemic was related to perceived health threat. There has also been a change in key factors in customers’ transportation choices, shifting from traditional cost and convenience to health and safety related factors. As a result, ‘reducing the risk of infection’ is now the primary factor in people’s choice of transportation mode. Therefore, shared mobility modes can benefit VSGs with more accessible transportation services than private cars and in some cases PT by providing precautionary measures during the pandemic such as distributing disinfectants to help drivers to keep cars clean, installing protective plastic sheets, reducing their fares during the pandemic, and disinfecting all high contact surfaces on bikes and scooters in respective depots. All these will increase the running cost for these companies that should be covered by the government through incentives and tax exemptions. The effectiveness of such measures in changing VSGs’ attitudes (scared to be infected) towards shared mobility modes can be a potential research topic.

Overall, it can be concluded that the COVID-19 pandemic has an unequal impact on different social and demographic groups in terms of mobility, in particular in different countries. The pandemic has proven a lack of inclusivity in transportation, particularly for the elderly and disabled commuters. This provides opportunity to ensure policies are in place to support the equity and inclusivity of transport infrastructure. For instance, subsidizing transportation, dedicating seats and spaces in public transport systems, and enhancing public awareness of the specific needs and rights of these vulnerable groups are important steps to achieve more inclusive transportation. Furthermore, since we are still struggling with the health-related concerns associated with the pandemic, policymakers and governments should still encourage safety measures (e.g., face covering and basic hygiene) for public transport users to reduce the spread of the virus.

In addition, in order to minimize future disruptions to the supply and demand dynamics, policymakers should invest more on travel demand management (TDM) plans. This would enable governments and transport providers to alleviate congestion and increase existing infrastructure capacity.

Most of the relevant studies have been in developed countries, while limited studies have been done regarding VSGs in South and Central America, Middle East, and Africa, in particular in low- and middle-income countries. However, it should be mentioned that we only reviewed articles that were written in English which may be a factor to the lack of information about Latin America or other parts of the world where English is not the main language. Hence, one should study the impact of COVID-19 on mobility of VSGs in Latin American, African, and Middle Eastern countries and include research published in languages other than English. A post-COVID-19 study could notably explore the impacts of tele-working and economic crisis on the drop-in demand for commuting transport by VSGs. The shift to fully or partly tele-working by companies and organizations, its benefits for VSGs in particular for persons with disabilities, and women can be other topics that requires further study.

## Figures and Tables

**Table 1:** Reviewed studies related to COVID-19 impacts on the mobility and activities of older people.

Study	Country	Mode	Main Findings
Beck & Hensher, 2020 [15]	Australia	Car	Older households made significantly less trips than younger households before and during the pandemic. Before the pandemic, older people were less concerned about the hygiene on public transit, but during the pandemic became as concerned as the other age groups. Older people were more likely to decrease the use of a car during the pandemic.
Daoust, 2020 [20]	Worldwide (27 countries)	-	Older people were more likely to avoid crowded places (e.g., public transport, gatherings), but were less compliant to wear a mask (degree of compliance for 20 year-old person is 0.6, whereas degree of compliance for 80 year-old person is only 0.3) and were not significantly more likely to self-isolate than other age groups despite their vulnerability to the virus.
de Haas et al., 2020 [7]	Netherlands	PT, car, bicycle and walk	The majority of older people (χ^2^ = 95.2 (1, *N* = 24920), *p* = 0.001) were much less active than before COVID-19 compared to other age groups for activities such as grocery shopping, shopping, exercising, and physical meetings. Sample size: 2500 respondents from the Netherlands Mobility Panel (MPN)
Heiler et al., 2020 [26]	Austria	-	Older people were less compliant to mobility restriction than the other age groups despite their vulnerability to COVID-19.
Kabiri et al., 2020 [28]	US	-	Older people were quick in accepting the stay-at-home measure, changing their behavior and practicing social distancing compared to other generations.
Oliver et al., 2020 [31]	Spain	-	Older people were more likely to stay at home (14.9%) compared to younger generations (7.6%).
Pant & Subedi, 2020 [32]	US	-	COVID precaution measures such as the stay-at-home measure increased the social isolation for all age groups, in particular older people. As a result, they could not meet their relatives and friends.
Pullano et al., 2020 [30]	France	-	Older people almost stopped taking trips longer than 100 km and were likely to avoid leisure activities and family trips.
Ragland et al., 2020 [29]	US	PT, car, ridesharing, special transportation service	In California between 2018 and 2020, for the age group 55 years and older, PT use decreased by 28.3%, special transportation services use increased by 2.9%, and ridesharing (only +65 years old) increased by slightly more than 10%. A small percentage of older people (3.7%) had a person to drive them to work before COVID-19 and this practice was no longer used in 2020. Older people changed home-to-work transport mode; a shift was mainly toward private cars (87.1% to 93.7%).
Yamada et al., 2020 [19]	Japan	Walk	In Japan due to COVID restrictions from 1 January to 25 May 2020, daily time spent in common areas and walking distance in care retirement communities decreased from 94 min/day to less than 80 min/day and 1300 m/day to approximately 900 m/day, respectively.
Stavrinos et al., 2020 [33]	US	Car	Post-COVID, both vehicle miles driven and driving days per week decreased by 35% and 37%, respectively. However, older adolescents, employed adolescents, and ethnic minorities were less likely to decrease their driving during the COVID-19 restriction period.
Leppä et al., 2021 [34]	Finland	Walk	During social distancing, older respondents with no walking difficulties were able to partly compensate for their decreased social life activities and interactions by increasing their physical activities (5.5 min/day, SD 25.1). They also faced less steep decline in their life-space mobility compared to those older respondents with walking difficulties.
Rantanen et al., 2021 [35]	Finland	Walk	During the COVID-19 outbreak, older people’s active aging scores (age and sex adjusted within subject B −24.1, SE 0.88, *p* < 0.001; η2 for time 0.508), as well as their life-space mobility score (B −10.8, SE 0.75, *p* < 0.001; η2 0.193, correspondingly), faced a steep decrease compared to the data from two years prior to the pandemic.
Eisenmann et al., 2021 [36]	Germany	Car ownership	Younger people had higher tendency to miss having a car of their own compared to older respondents. These respondents were mainly women between the age of 18 and 44 who used public transport as their main means of transport during the lockdown restrictions and perceived inconvenience with the use of public transport.
Chan et al., 2021 [37]	Worldwide (31 countries)	-	The age of respondents was found to be influential in their compliance to reduce their mobility and stay at home. Both older and younger respondents (compared to middle-aged [30–60 years old]) were more likely to stay at home during lockdown restrictions.
Rahimi et al., 2021 [38]	US	shared mobility	Concerning age, older respondents perceived a higher risk than younger respondents regarding using shared mobility services.
Pawar et al., 2021 [16]	India	-	Age was found to be a critical factor affecting the travel frequency of work-based trips, where younger commuters were found to be more likely to shift to no travel during the transition to lockdown restrictions. The analysis indicated that for each year increase in the age of travelers, their probability of no travel during the travel restriction would decrease by 2 percent.
Fu and Zhai, 2021 [39]	US	-	Due to their dependence on the assistance of others, older respondents (aged 65 and older) generally had less compliance with social distancing and stay-at-home behaviors.
Lee et al., 2021 [40]	South Korea	-	The average non-home trips and activities for the older people was higher compared to non-older people, whereas the average non-home activity time per person for the older people was about 2 h and 10 min shorter. Furthermore, on average, the older people had slightly higher number of trips compared to non-older people (4.90 trips/person and 4.74 trips/person, respectively). People aged over 80 spent the longest time at home (average of 16.74 h) compared to people in their 30s that stayed at home for the shortest amount of time (average of 13.24 h).

**Table 2:** Reviewed studies related to COVID-19 impacts on the mobility and activities of disabled persons.

Study	Country	Mode	Disability Type	Main Findings
Ainslie, 2020 [41]	UK	-	Mental impairments, hearing impairments, mobility impairments.	PwDs were more likely to leave their home for medical purposes and to provide help to a vulnerable person than the rest of the population (19% against 7%). However, they were less likely to leave their home for leisure, to commute, to take the children to school, to grocery shop, to exercise, or to meet up with people. In the data collected in May 2020, 73.4% of PwDs left their home against 92.5% of non-disabled persons to do their regular activities. Among PwDs, 80% of persons with mental impairments left their home against 54.7% for persons with hearing impairments and 65.9% of persons with mobility impairments.
Beukenhorst et al., 2020 [42]	US	-	Amyotrophic lateral sclerosis, a kind of mobility impairment	During the COVID-19 pandemic, the median time spent at home for amyotrophic lateral sclerosis (ALS) people increased from 19.4 h to almost 23.7 h, and the median daily distance travelled dropped from 42 km to 3.7 km. For general population in the US, daily time spent home increased from 10 to 14 h.
Chen et al., 2020 [25]	US	Paratransit	-	Many PwDs rely on paratransit, such as a minibus (or van) equipped with wheelchair lifts or ramps to facilitate access. Paratransit use dropped during the beginning of the pandemic by around 80% but recovered to 50% of the normal service in late July 2020. A total of 47% of 2000 PwDs who participated in the survey rely on personal care, in which 27% of them (216 persons) stopped receiving those services during the outbreak.
Cochran, 2020 [21]	US	PT and ride hailing (Uber, etc.)	Visual impairments: blind or low visibility, hearing impairments; other disabilities	The pandemic aggravated the difficulties of PwDs to access PT and created more reluctance to use them. PwDs experienced less assistance in accessing PT and completing daily living activities than prior to the COVID-19 pandemic. PwDs were worried about being infected from people (drivers/passengers) or surfaces (transportation facilities/vehicles), e.g., blind persons should touch surfaces for navigation while using PT or ride haling services. Blind persons could neither get accurate and timely information about transport service, such as whether or not it was operating, nor up to date information about spread of COVID-19 in their region. Then, it was difficult for them to evaluate the risk of travelling by PT or ride hailing services.
Eskytė et al., 2020 [22]	UK	Walk	All forms of impairments	- Stay-at-home measure: A substantial number of PwDs were no longer receiving healthcare visits at home and assistance with shopping. - Physical distancing and use of face mask: People with hearing impairments could not communicate with fellow pedestrians due to fellow pedestrians using non-transparent face masks. The 2-m physical distancing while walking was difficult to follow for persons with visual/mobility/cognitive impairments or neurodiversity in non-disability-inclusive sidewalks.
RIDC, 2020a, 2020b	UK	PT	-	Travel by PT was dropped significantly for most PWDs (64% of respondents) due to safety concern, a lack of trust with the information provided by the government, and a heightened feeling of vulnerability to COVID-19. In addition, 50% of respondents were no receiving health, personal care, as well as shopping assistant during the pandemic.
Schur et al., 2020 [43]	US	-	-	Tele-working due to the COVID-19 pandemic positively influenced the employment opportunities for people with disabilities, but there is still a wage gap between non-disabled and disabled people. Increased availability of home-based work in the future can create more employment opportunities for people with disabilities.
Leppä et al., 2021 [34]	Finland	Walk	-	Life-space mobility for older respondents with impaired walking decreased significantly, putting them at risk of being housebound (*p* = 0.001). Furthermore, respondents with impaired walking had a smaller decrease in autonomy of participating in outdoor activities (*p* = 0.017) and slighter increase in their physical activity (*p* < 0.001) compared to those with intact walking ability.
Rahimi et al., 2021 [38]	U.S.	shared mobility	-	Respondents’ health background, such as their pre-existing health conditions and disability status, significantly influenced their risk perception associated with the usage of public transport.
Fu and Zhai, 2021 [39]	U.S.	-	-	At the beginning of lockdown restrictions, people with disability were mainly staying at home due to their special needs and reliance on assistance of others. However, throughout the lockdown period and with the growth of the pandemic situation, disabled people generally decreased their social distancing and stay-at-home behaviors as they needed to take care of themselves or were dependent on support from other community members.

**Table 3:** Reviewed studies related to COVID-19 impacts on the mobility and activities of different genders.

Study	Country	Mode	Main Findings
Abdullah et al., 2020 [6]	Worldwide	-	Pre-COVID pandemic, mode choice for primary trips purposes were similar for females and males. Males used private transport modes at a higher rate and undertook more and longer trips during COVID-19 (+3.9% and +1%) compared to females (−9% and −2%), whereas females were not likely to change their mode choice. Females might be more concerned about being infected during the pandemic.
Assoum ou et al., 2020 [24]	Belgium	PT	Considering jobs (caregiving, primary and pre-primary education, housework, and domestic work) held by females among the working-age (20–59 years old) population in Belgium, females were more vulnerable to be infected during lockdown as they had frontline jobs and their main transport mode was PT. The female/male COVID cases index confirmed this vulnerability.
Bhaduri et al., 2020 [44]	India	-	Females’ work and discretionary activities decreased more than males during the COVID-19 pandemic (a decrease of 17% of work activities compared to 9% for males and decrease of 34% of females’ discretionary activities against 28% for males). The shift to home-based work was slightly higher for females (+19%) than males (+16%). Females have less tendency to use a car to commute (because of less driver’s license ownership compared to males) but had more tendency to work from home and to use other means of transport.
Beck & Hensher, 2020 [15]	Australia	Car and PT	Females’ concern about levels of hygiene on PT was similar before and after COVID-19. Females’ activities such as shopping, visiting relatives and friends, and healthcare appointments have been interrupted due to COVID-19.
Chen et al., 2020 [25]	US	-	During the pandemic, pregnant females were less willing to travel outside their home for prenatal care (usually not amenable to telemedicine). Females had disproportionately more childcare obligation and were more impacted than males by school closures.
Matson et al., 2021 [5]	US	-	The attitude toward tele-working is different between females depending on the presence of children in the household. Working mothers stated there are unwanted distractions while tele-working.
Dandap at et al., 2020 [23]	India	-	Males had less tendency to work from home than females.
Heiler et al., 2020 [26]	Austria	-	Mobility behaviour of the male worker population changed more significantly than females, possibly explained by the obligation of home office work.
Molloy, 2020 [12]	Switzerland	-	During lockdown, males travelled longer distances. Average daily distances travelled had a more consequent drop for females (from 38 km prior COVID-19 to 12.5 km at the beginning of the lockdown, recovered at 37 km in late August 2020) than for males (46 km, 18 km, and 46 km, respectively).
Shakibaei et al., 2020 [10]	Turkey	Rail transit and Car	Females were used to rail transit more than males pre-, during, and post-lockdown. However, females’ travel behaviour changed during the outbreak. This shows rail transit was more reliable and secure for females compared to road transportation. An increase in females’ car use to commute (home to work trips) was observed, but still they used a car less than males, and grocery shopped less than males.
Thombre & Agarwal, 2020 [11]	India	-	In India pre-lockdown, PT and walking were the most preferred modes among females. After the outbreak, the share of PT decreased (27% to 22%), the share of walking travels slightly increased (27% to 29%), and the share of motorized vehicle significantly increased (18% to 25% in the five biggest megacities). After the lockdown, non-motorized transports (NMT) and intermediate public transport (IPT) decreased (24% to 15%). The findings highlight the importance of PT, NMT, and IPT modes to ensure gender equity.
Eisenma nn et al., 2021 [36]	Germany	Bicycle/car ownership/PT	Bicycle usage decreased more sharply for males (minus 10 percentage points) than for females (minus 5 percentage points). Public transport usage dropped more significantly amongst males (from 22% to 10%) than amongst females (from 24% to 15%). The regression model indicates that females were more likely than males to miss having a car of their own.
Chan et al., 2021 [37]	Worldwide	-	Concerning gender, women (compared to men) were more compliant and cooperative to stay at home previously (b ¼ 0.037, SE ¼ 0.015) and continue to stay at home in the future (OR ¼ 0.79, SE ¼ 0.044). They were also more likely to reduce their mobility during lockdown restrictions.
Rahimi et al., 2021 [38]	US	shared mobility	Gender of respondents had a significant role on their perceived risk of using shared mobility services during the pandemic. Females perceived higher risks of using shared mobility modes.
Lee et al., 2021 [40]	South Korea	-	In terms of activity behaviors by gender, women, regardless of their age group, had longer duration of home activity time than men. For instance, the average home activity time for both non-older and older women (14.51 h and 16.76 h, respectively) is longer than those of non-older and older men (12.68 h and 14.83 h, respectively). The average home activity times were 12.68 h for non-older men, 14.51 h for non-older women, 14.83 h for older men, and 16.76 h for older women. On average, men tend to participate in more non-home activities per day compared to women. On average, women have shorter non-home activities (9.41 h) compared to men (11.20 h).
Politis et al., 2021 [45]	Greece	-	In terms of both travel duration and trip frequencies, men tended to make longer and more trips during lockdown restrictions compared to their women counterparts. Men had a hazard ratio of 0.90, which indicated that the duration of travel for male travelers was somewhat longer compared to female travelers. In general, trips made by walking and cycling were likely to be shorter than the trips made by cars (hazard ratios of 1.49 and 1.94, respectively). Moreover, trips made by public transport were more likely to have a significantly longer duration compared to cars (hazard ratio of 0.56).

**Table 4:** Reviewed studies related to COVID-19 impacts on the mobility and activities of low-income peoples.

Study	Country	Mode	Main Findings
Bert et al., 2020 [52]	Worldwide (China, EU, US)	Privat e car,	In post-lockdown, MIP (middle-income population) were slightly more willing to buy a car compared to LIP in the U.S., while in the EU, all income groups had similar likelihood to buy a new car post-lockdown. In China, LIP were less likely to buy a new car post-lockdown compared to MIP and HIP.
Beck & Hensher, 2020 [15]	Australia	Car and PT	Pre-COVID, LIP made significantly less trips per week compared to other income groups, while post COVID, there was no difference between income groups in terms of the number of trips. As most of the LIP were less likely to own a car, they have shown significantly lower average car use reduction compared to HIP. LIP were less likely to do work from home compared to MIP and HIP. As expected, LIP were less likely to show reduction in some activities such as going to restaurants, cafés, pubs or bars, gyms or exercise, watching professional sport, playing organised sports, or work functions.
Bhaduri et al., 2020 [44]	India	-	In terms of working habits during the pandemic, LIP reduced working much more than HIP (−29% compared to −1%), possibly due to their lower tendency to telecommute and their lower rate of car ownership. HIP were more likely to shift to work from home than LIP (+20% and +11%, respectively).
Dandapat et al., 2020 [23]	India	PT	Mostly being from low-income groups, captive riders to PT are more likely to use PT during the pandemic despite their concern about the risk of infection.
Hernando et al., 2020 [48]	Spain	-	Means of the daily radius of gyration collected using mobile phone data has been used as a measure to evaluate the mobility inequality across the Spanish population: LIP: pre-lockdown (former lockdown): 8.1 km, 3.3 km in lockdown, 6.9 km after lockdown (new normal). HIP: pre-lockdown (former lockdown): 6.9 km, 0.9 km during lockdown, 4.7 km after lockdown (new normal). In former normal (pre-lockdown), the mean radius of gyration for LIP and HIP were 8.1 and 6.9 km which shows 17% mobility inequality. As most of LIP jobs are not suitable for teleworking, inequality sharply increased from 17% to 47% due to teleworking in new normal (post- lockdown).
Koehl, 2020 [51]	UK	-	Increasing the share of active transportation (cycling and walking), as also suggested by the WHO during the COVID-19 pandemic, can decrease the pressure on the often-overloaded PT systems which is the most used transport modes in LIP and MIP countries.
Ramit et al., 2020 [49]	India	All modes	Only a 23% shift was expected for the intra-city urban rail used in Mumbai and Chennai post-lockdown as LIP (income < 25,000 INR) who do not own a vehicle were the highest portion among PT users. Regardless the income, only 24% of respondents were more likely to buy a new vehicle post lockdown. Among them, LIP and MIP (25,000 < income < 50,000 INR) were most likely to by a two-wheeler.
Ruiz-euler et al., 2020 [47]	US	-	Lockdown policies increased the mobility gap (differences in mobility across income levels) and inequality in urban centres of American cities. LIP were unable to reduce mobility (distance travelled) as much as high-income groups during the outbreak.
Thombre & Agarwal, 2020 [11]	India	All modes	Before the lockdown, the preferred modes of LIP for primary activities in megacities were PT, walking, and motorized two-wheeler, respectively. In post-lockdown, PT and walking trips sharply decreased among LIP, and they made a shift to motorized transports for LIP, possibly explained by a higher preference to safety than affordability.
Tirachini et al., 2020 [53]	Chile	-	Low-income workers were less (1 out of 4 workers) able to work from home.
Lou et al., 2020 [54]	Worldwide	-	The “stay-at-home” measure has less effect on LIP’s mobility than higher income groups’ mobility. Work and non-work-related trips were less reduced for LIP as more essential jobs were held by LIP as they could not afford online shopping or do tele-working. This difference in mobility is less significant in sparsely populated regions.
Pawar et al., 2021 [55]	India	-	Higher income groups were less likely (approximately 14–25% reduction in chances of having reduced travel) to switch to no travel compared to LIP. All income groups were likely to significantly reduce their non-work-based trips, but higher income groups were more likely to travel regularly for non-work-related purpose than LIP.
Iio et al., 2021 [56]	US	-	Before COVID restrictions, the distance travelled by income groups were similar. During the pandemic (in April 2020), the median monthly distance travelled by high-income groups had a large decrease compared to LIP. The radius of gyration and the number of locations visited dropped in a larger proportion for higher income groups than that for LIP.
Matson et al., 2021a [5]	US	-	LIP are less likely to work from home and benefit from the ensuing travel time savings; therefore, a long-term shift toward tele-working may increase the current mobility inequities.
Matson et al., 2021b [50]	US	Ride-hailing	The use of ride hailing services for LIP does not change pre- and during the COVID outbreak but the change for HIP and MIP was obvious.
DfT, 2021 [57]	UK	-	LIP travelled less than high-and middle-income respondents, while high-income groups had similar travel pattern such as before the pandemic.
Rahimi et al., 2021 [38]	US	shared mobility	People’s income was found to play a critical role on their risk-perception behavior associated with shared mobility services. According to the result, those respondents from extremely low-income background (with less than $20 K income per year) perceived higher risks of exposure to COVID-19 associated with the use of public transport modes.
Pawar et al., 2021 [55]	India	-	In terms of the effect of travelers’ income on their work-and non-work-related travel frequency, travelers from higher-income brackets (3 to 6 lakh rupees or 6 to 12 lakh rupees) were significantly less likely to opt to no travel during the transition to lockdown period compared to lower income travelers (up to 3 lakh rupees). The findings revealed that the chances of switching to ‘no travel’ decreased by 61% and 45% for travelers with income of 6–12 lakhs and 3–6 lakhs, respectively, compared to those with income less than 3 lakh rupees (1 lakh = 0.1 million).

## Data Availability

Not applicable.

## Appendix A

Table A1 lists the details (i.e., thematic focus, authors, year, country, social group examined) of the selected papers for our analysis. We did not filter the journal names in our search process (PRISMA method), so that our search includes all journals which have studies on COVID-19 and VSGs.

**Table A1:** The profile of the studies selected for the literature review.

#	Thematic Focus	Authors	Study Area	Year	People on Low Income	Females	Older People	People with Disabilities
1	Public transit; carpooling; ride-hailing and taxi; car- haling; micro mobility sharing; bike; walk; private car	Bert et al.	Worldwide	2020		-	-	-
2	Commuting behaviour	Tirachini et al.	Chile	2020		-	-	-
3	Distance	Ruiz-Euler et al.	US	2020		-	-	-
4	Travel satisfaction	Khaddar & Fatmi	Canada	2020		-	-	-
5	Transportation policies in low- and middle-income countries	Koehl	Worldwide	2020		-	-	-
6	Vehicle ownership, mode share, willingness to buy a new vehicle	Ramit et al.	India	2020		-	-	-
7	Mode share, trip motives	Shamshiripour et al.	US	2020		-	-	-
8	Mode share	Meena	India	2020		-	-	-
9	Mean radius of gyration	Hernando et al.	Spain	2020		-	-	-
10	Impacts on trip purposes	Lou et al.	Worldwide	2020		-	-	-
11	Travel demand	Circella	US	2020		-	-	-
12	Travel demand	Jay et al.	US	2020		-	-	-
13	Radii of gyration; travel distance; frequency of travel	Iio et al.	US	2021		-	-	-
14	Travel behaviour: distance travelled; work and non-work-related travel frequency	Kar et al.	US	2021		-	-	-
15	Ride-hailing	Matson et al.	US	2021		-	-	-
16	Trip motives	Assoumou Ella,	Belgium	2020	-		-	-
17	Trip purpose; mode choice; distance travelled; and frequency of trips before and during COVID-19.	Abdullah et al.	Worldwide	2020	-		-	-
18	Changes in the share of travel modes	Shakibaei et al.	Turkey	2020	-		-	-
19	Walking distance, daily time spent in common areas	Yamada et al.	Japan	2020	-	-		-
20	Activity time	Rantanen et al.	Finland	2020	-	-		-
21	Modal share changes during COVID	Ragland et al.	US	2020	-	-		-
22	Social isolation	Pant & Subedi	Worldwide	2020	-	-		-
23	Travel motives	Oliver et al.	Spain	2020	-	-		-
24	Behavioural changes: mask wearing, PT avoidance, guest avoidance	Daoust	Worldwide	2020	-	-		-
25	Ridership, distances travelled	Kabiri et al.	US	2020	-	-		-
26	Ridership, distances travelled	Pullano et al.	France	2020	-	-		-
27	Car; driving behaviour	Stavrinos et al.	US	2020				-
28	Outdoor activities; working from home; home education; share of travel mode; share of trip motives	de Haas et al.	Netherland	2020	-	-		-
29	Private car; public transport; ride hailing/sharing; ferry; train; walk; bicycle; trip motives	Beck & Hensher	Australia	2020				-
30	life-space mobility; active aging; walk	Rantanen et al.	Finland	2021	-	-		-
31	Well-being and travel behaviour	Ainslie	UK	2020	-	-	-	
32	Street time occupancy	Eskytė et al.	UK	2020	-	-	-	
33	COVID and access on transportation	Cochran	US	2020	-	-	-	
34	Daily home time and daily distance travelled	Beukenhorst et al.	US	2020	-	-	-	
35	Commuting for PwDs	Schur et al.	US	2020	-	-	-	
36	PT, Private car, walk, bicycle	Thombre & Agarwal	India	2020			-	-
37	Changes in travel characteristics, perceived risk of different modes, mode preference after the pandemic	Dandapat et al.	India	2020			-	-
38	Trip length and motives:	Bhaduri et al.	India	2020			-	-
39	Private car; public transit; paratransit, transportation network companies; non- emergency medical transportation; walk and bicycle	Chen et al.	US	2020			-	
40	Telecommuting Rates During the Pandemic	Matson et al.	US	2021			-	-
41	Mobile phone data	Heiler et al.	Austria	2020				-
42	Distance; active days; modal share; trip motives	Molloy	Switzerland	2020				-
43	Public transport; car ownership	Eisenmann et al.	Germany	2021				-
44	Activity and travel patterns	Lee et al.	South Korea	2021				-
45	Traits and regulatory compliance during COVID lockdown; mobility behaviour; willingness to reduce outdoor mobility	Chan et al.	Worldwide	2021				-
46	Life-space mobility; Autonomy in participation outdoor physical activities; walk	Leppä et al.	Finland	2021				
47	Shared mobility services	Rahimi et al.	US	2021				
48	Work-and non-work-based trip patterns	Pawar et al.	India	2021				-
49	Social vulnerability and stay-at-home behaviour	Fu & Zhai	US	2021				
50	Travel behaviour patterns	Politis et al.	Greece	2021				-
